# Liver development is restored by blastocyst complementation of *HHEX* knockout in mice and pigs

**DOI:** 10.1186/s13287-021-02348-z

**Published:** 2021-05-19

**Authors:** M. Ruiz-Estevez, A. T. Crane, P. Rodriguez-Villamil, F. L. Ongaratto, Yun You, A. R. Steevens, C. Hill, T. Goldsmith, D. A. Webster, L. Sherry, S. Lim, N. Denman, W. C. Low, D. F. Carlson, J. R. Dutton, C. J. Steer, O. Gafni

**Affiliations:** 1 Recombinetics Inc., Stem Cell Technologies, 3388 Mike Collins Drive, Eagan, MN 55121 USA; 2 Department of Neurosurgery, University of Minnesota, Minneapolis, USA; 3 Stem Cell Institute, University of Minnesota, Minneapolis, USA; 4 Mouse Genetics Laboratory, University of Minnesota, Minneapolis, USA; 5 Bioinformatics and Computational Biology Program, University of Minnesota, Minneapolis, USA; 6 Department of Genetics, Cell Biology and Development, University of Minnesota, Minneapolis, USA; 7 Department of Medicine, University of Minnesota, 420 Delaware Street SE, MMC 36, Minneapolis, MN 55455 USA

**Keywords:** Development, Stem cells, Gene editing, Embryo, Transplantation

## Abstract

**Background:**

There are over 17,000 patients in the US waiting to receive liver transplants, and these numbers are increasing dramatically. Significant effort is being made to obtain functional hepatocytes and liver tissue that can for therapeutic use in patients. Blastocyst complementation is a challenging, innovative technology that could fundamentally change the future of organ transplantation. It requires the knockout (KO) of genes essential for cell or organ development in early stage host embryos followed by injection of donor pluripotent stem cells (PSCs) into host blastocysts to generate chimeric offspring in which progeny of the donor cells populate the open niche to develop functional tissues and organs.

**Methods:**

The *HHEX* gene is necessary for proper liver development. We engineered loss of *HHEX* gene expression in early mouse and pig embryos and performed intraspecies blastocyst complementation of *HHEX* KO embryos with eGFP-labeled PSCs in order to rescue the loss of liver development.

**Results:**

Loss of *HHEX* gene expression resulted in embryonic lethality at day 10.5 in mice and produced characteristics of lethality at day 18 in pigs, with absence of liver tissue in both species. Analyses of mouse and pig *HHEX* KO fetuses confirmed significant loss of liver-specific gene and protein expression. Intraspecies blastocyst complementation restored liver formation and liver-specific proteins in both mouse and pig. Livers in complemented chimeric fetuses in both species were comprised of eGFP-labeled donor-derived cells and survived beyond the previously observed time of *HHEX* KO embryonic lethality.

**Conclusions:**

This work demonstrates that loss of liver development in the *HHEX* KO can be rescued via blastocyst complementation in both mice and pigs. This complementation strategy is the first step towards generating interspecies chimeras for the goal of producing human liver cells, tissues, and potentially complete organs for clinical transplantation.

**Supplementary Information:**

The online version contains supplementary material available at 10.1186/s13287-021-02348-z.

## Background

There are currently more than 17,000 people in the US waiting to receive liver transplants. These numbers are steadily increasing because of two major factors: the rapidly climbing age of the US population and the rising incidences of chronic liver diseases, specifically alpha-1 antitrypsin deficiency, alcoholic liver disease, hepatitis B and C, and most notably, Nonalcoholic steatohepatitis (NASH). As a result, there is an enormous effort to obtain functional liver tissues and hepatocytes that can be used to restore liver function in patients. Multiple in vitro hepatocyte differentiation protocols using human induced pluripotent stem cells (hiPSCs) have been published [1, 2], yet applying these differentiated cells in effective treatments has not been achieved. Several reasons include significant discrepancies in protein expression profiles and epigenetic landscape between in vitro and in vivo differentiated liver cells, low scalability, and poor long-term cell function after transplantation [3, 4]. Other studies have attempted using hiPSCs to generate functional full-size livers utilizing decellularized scaffolds [5], liver spheroids by three-dimensional bioprinting [6], or hepatocyte-like organoids [7]. However, it is challenging to scale these in vitro programs into a functional liver.

Blastocyst complementation may allow the use of animals as bio-incubators to grow cells, tissues, and even complete organs from one species within the body of a different, but physiologically similar species. This technology, originally proposed by Chen et al. [8] with a resurgence by Kobayashi et al. [9], requires the deletion of specific gene(s) that are essential for the early development of a tissue or organ. This is followed by microinjection of donor PSCs or lineage-specific progenitor cells into the host blastocyst to generate chimeric offspring in which cells that are the progeny of the donor cells populate the open niche. Blastocyst complementation to generate pancreatic tissue has been explored extensively and has demonstrated the feasibility of intraspecies chimeras in a livestock animal [10, 11] as well as interspecies chimeras, especially between phylogenetically similar species, such as mice and rats [9, 12, 13]. In one of those studies, pancreatic islets derived from donor PSCs were capable of responding to blood glucose fluctuations in mice or rats after allogeneic transplantation. Other studies have used this technology to create organs such as kidney, brain, lungs, vessels, blood, and liver [10, 14–19], using pigs as bio-incubators of primate tissues in some cases [20–22].

The *HHEX* gene encodes a hematopoietically expressed homeobox protein highly conserved among mouse and human [23] with orthologs in all metazoan species examined to date. It functions as a transcription factor both activating and repressing other developmental genes and is one of the earliest markers of the anterior endoderm, which develops into the foregut and gives rise to organs such as the liver, ventral pancreas, thyroid, and lungs [24–26]. Previous studies have shown that *HHEX* is required for proper liver development [27–29]. In the absence of *HHEX* expression, cells from the liver bud (formed between E8.5–E9.5 in mice) cannot transition from columnar to pseudostratified epithelium, preventing migration of the hepatoblasts to the septum transversum and blocking consequent liver differentiation [30]. Hunter et al. [31] found that *HHEX* is also required in multiple steps in the development of the hepatobiliary system development after liver bud formation. When *HHEX* expression was suppressed at different time points, various phenotypes appeared including abnormal morphogenesis of the intrahepatic bile ducts, severely hypoplastic and cystic liver, and defects of the extrahepatic biliary tract and hepatic epithelium. Due to the cardinal role of *HHEX* affecting several different key developmental pathways, the null mutation of this gene results in embryonic lethality in mice around E10.5–E11.5 [32, 33].

Forebrain development is also dependent on *HHEX* expression. Barbera et al. [27] described three different *Hhex* KO phenotypes in mice based on the extent of forebrain defects. *HHEX* is also a transcriptional regulator of the VEGFC/FLT4/PROX1 signaling axis during angiogenic sprouting and lymphatic formation in vertebrates. In E10.5 *Hhex* KO mice, *Flt4* expression is reduced in the intersomitic vessels, causing pericardial edemas as well as developmental delays [33]. Other studies have shown that *HHEX* plays a critical role in heart and vascular development as *Hhex*-null mutation resulted in abnormal cardiac formation, defective vasculogenesis, and elevated VEGFA levels [34, 35].

In the current study, we have generated CRISPR/Cas 9-mediated *HHEX* knockouts in mouse and pig embryos. *HHEX* KO in both species was embryonic lethal and resulted in retarded development. Gross morphological and molecular phenotyping in both species demonstrated ahepatogenesis and dysregulation of developmental genes in multiple tissue systems. Using intraspecies blastocyst complementation we were then able to rescue liver development in both mouse and pig *HHEX* KO embryos.

## Methods

### Mouse zygote isolation and *Hhex* knockout

Briefly, 3.5-week-old female C57BL6/J mice (Jackson Laboratory, Bar Harbor, ME) were superovulated using pregnant mare’s serum gonadotropin (5 IU i.p.; National Hormone and Peptide Program, Harbor-UCLA Medical Center, Torrance, CA) at 1:00 p.m. followed 47.5 h later with human chorionic gonadotropin (5 IU i.p.; National Hormone and Peptide Program) and immediately mated with C57BL6/J 4-month-old stud males (Jackson Laboratory, Bar Harbor, ME). The following morning, female mice were observed for the presence of vaginal plugs, and this was considered embryonic day 0.5 (E0.5). Following cervical dislocation, the ovary, oviduct, and proximal end of the uterine horn was dissected from both sides of female mice and placed in a drop of modified Human Tubal Fluid media (mHTF) [36] (#90126, Irvine Scientific, Santa Clara, CA). Zygotes were extracted from the oviduct of all mice and pooled together in 500 μl of 0.3 mg/ml in hyaluronidase in mHTF (#H4272, Sigma-Aldrich, St. Louis, MO) and placed in the incubator (37 °C and 5% CO_2_) for 2 min to remove the zygotes from the cumulus-oocyte complex. The zygotes were washed twice in Opti-MEM (#31985062, Thermo Fisher Scientific, Waltham, MA) and transferred to a pre-chilled (4 °C) 1 mm electroporation cuvette (#5510-11, MBP Inc., Toronto, Canada) containing the electroporation mixture in Opti-MEM: 250 ng/μl of duplexed tracrRNA with mmHHEX gRNA2.1 (CCACAGGCAAGCCCUUGCUC; Synthego, Redwood City, CA; Supplemental Figure S1a) and 250 ng/μl Cas9 (#1081060, Integrated DNA Technologies, Inc., Coralville, IA) in a final volume of 30 μl. The cuvette was inserted in the Gene Pulser Xcell electroporator (Bio-Rad, Hercules, CA) and the zygotes were electroporated using the following parameters: two square wave pulses at 30 V and 3 ms duration with a 100 ms interval. After retrieving the zygotes from the cuvette, they were washed twice in mHTF and transferred to a drop of mHTF under mineral oil and placed in the incubator (37 °C, 5% CO_2_) until they reached the blastocyst stage (E3.5), approximately 72 h later. Blastocysts were then transferred around 5 p.m. into the uteri of Avertin anesthetized (225 mg/kg) 6-week-old CD1 pseudo-pregnant female mice (22–24 g; Charles River, Wilmington, MA) 2 days following the presence of vaginal plug (E2.5) post-mating with 4-month-old vasectomized CD1 male mice (Charles River). For all subsequent analyses, the indicated age of the post-implantation embryo was matched to the time-mated CD1 female.

### Mouse blastocyst injection and embryo transfer

For blastocyst complementation, eGFP-labeled single-cell mouse induced pluripotent stem cells (miPSCs) from the *UMN-3F10* line [37] were suspended with E3.5 mouse blastocysts in a drop of EmbryoMax M2 media (#MR-015-D, Millipore Sigma, Burlington, MA), and ~ 10 miPSCs were injected into the blastocoel cavity near the inner cell mass of all blastocysts electroporated as mentioned above, regardless of genotype. After blastocyst injection, embryos were returned to mHTF drops under mineral oil, placed in the incubator (37 °C, 5% CO_2_), and cultured for 2–4 h. Injected blastocysts were then transferred into the uteri of E2.5-pseudo-pregnant female mice as described above.

### Production of the HHEX KO pigs by microinjection

In vitro fertilized (IVF) embryos (E0) (slaughterhouse oocytes with undefined breed and Yorkshire sperm) were washed in porcine zygote medium-3 (PZM-3) 5 h post-fertilization, denuded by pipetting and prepared for microinjection. Zygotes were incubated on a thermal plate at 38 °C for 5 min in microinjection medium composed of PZM-3, 0.4% BSA (#A9418, Sigma-Aldrich), and 5 μg/ml cytochalasin B (#C6762, Sigma-Aldrich) covered by mineral oil. Complexes of 100 ng/μl Cas9 protein, two sgRNAs 50 ng/μl each (ssHHEX gRNA 2.4: 5′-CAATGACCAGACCATTGAGC-3′; ssHHEX gRNA 2.5: 5′-AGGCAGGTGAGCTCGCTCAG-3′; Supplemental Figure S1b) and 50 ng/μl Stitch HDR oligo template to fuse the sequence after the deletion (all from Synthego, Redwood City, CA) were loaded into the microinjection needle using micro loader tips (#E5242956003, Eppendorf, Hamburg, Germany). The deletion created by the two guides was designed to result in a premature stop codon in the middle of exon 3. Then, needles were placed in a universal capillary holder controlled by a micromanipulator (Narishige International USA, Inc., Amityville, NY) coupled to a microinjection apparatus (Femtojet 4i Microinjector, Eppendorf). Microinjection volume was approximately 15 pl (1.5% of total embryo volume). Microinjected embryos were cultured in PZM-3 medium at 39 °C, 5% CO_2_, 5% O_2_, and 90% N_2_ under controlled atmosphere for 12 to 20 h until transfer at E1 to the uteri of synchronized 250- to 350-day-old Yorkshire gilts on day 1 of estrus (80–120 embryos per recipient).

### Somatic cell nuclear transfer (SCNT) in pig

SCNT was performed as described previously [38], using in vitro matured oocytes from slaughterhouse with undefined breed (DeSoto Biosciences, LLC, Seymour, TN). Cumulus cells were removed from the oocytes by pipetting in 0.1% hyaluronidase (#H3506, Sigma-Aldrich). Only oocytes with normal morphology and a visible polar body were selected for SCNT. Oocytes were incubated in manipulation media (PZM-3) containing 5 μg/ml bisbenzimide (Hoechst 33342; #B2261, Sigma-Aldrich) and 7.5 μg/ml cytochalasin B (# C6762, Sigma-Aldrich) for 15 min. Oocytes were enucleated by removing the first polar body plus metaphase II plate. Then, *HHEX* KO pig host embryos were created by injecting one *HHEX* KO porcine embryonic fibroblast (PEF) edited in exon 2 of the *HHEX* gene with TALENs (right arm: 5′-AGCCACTCTTGCCCAC-3′; left arm: 5′-TGGAGCCCCTTCCTTC-3′; HDR integration containing a stop codon with a HindIII site for diagnosis: 5′-TAAGCTT-3′; Supplemental Figure S1c; all from Synthego, Redwood City, CA) into an enucleated oocyte. Edition of the *HHEX* gene in this PEF cell line was confirmed by PCR followed by product digestion with HindIII (see “*HHEX* PCR genotyping” section in Supplemental for details). eGFP-labeled donor blastomeres were originated by injecting one eGFP-H2B PEF [39] into a different enucleated oocyte. The injections were followed by fusion and activation (considered E0) simultaneously by two DC pulses of 120 V for 30 μs (BTX cell electroporator; Harvard Apparatus, Holliston, MA) in 280 mM Mannitol (M4125, Sigma-Aldrich), 0.1 mM CaCl_2_ (#C4901, Sigma-Aldrich) and 0.05 mM MgCl_2_ (#M8266, Sigma-Aldrich). Activated embryos were placed back in PZM-3 medium with 0.4% bovine serum albumin (BSA) (#A9418, Sigma-Aldrich) and cultured at 39 °C, 5% CO_2_, 5% O_2_ in a humidified atmosphere. Presumptive embryos were cultured for 4.5 days before the complementation.

### Blastocyst complementation and embryo transfer in pig

The SCNT cloned embryos from both groups (eGFP^+^ and *HHEX* KO) were cultured in PorciPro In Vitro Culture media (MOFA Global, Verona, WI) for 4.5 days under mineral oil, and tri-gas incubator conditions (5% CO_2_, 5% O_2_, 90% N_2_, 38.5 °C, and high humidity). Using micromanipulation techniques, approximately 8–10 dissociated eGFP-labeled donor blastomeres were drawn into a glass pipette and inserted into the perivitelline space of SCNT cloned morula-stage E4.5 embryos. The E4.5 chimeric embryos were further cultured in PorciPro In Vitro Culture media (MOFA Global) in the same conditions as above, until they were transferred to the uteri of synchronized 250- to 350-day-old Yorkshire gilts on day 4.5 of estrus the same day.

### Preparation of complemented mouse embryo samples for next generation sequencing (NGS)

Two outcomes are of critical importance in the analysis of mouse embryos following intraspecies blastocyst complementation: determining the contribution of donor cells, and the genotype of the host embryo. eGFP fluorescence by donor cells confirms chimerism but it does not quantify the ratio of donor cells to host cells. Similarly, our genotyping protocol could not distinguish between chimeras in heterozygous or homozygous host embryos (all mouse embryos are injected with donor cells without prior knowledge of genotype).

As the donor cells were derived from C57BL/6 x 129Sv1 crosses and host embryos from C57BL6/J mice, we were able to identify two heterozygous single nucleotide polymorphisms (SNPs) in the *Olfr16* gene of the donor cells (G/A and C/T) not present in the host embryo (G/G and C/C). Therefore, quantification of NGS reads containing the SNPs of 129Sv1 strain (A and T) versus all reads was representative of the ratio of donor to host cells. For each complemented embryo, we amplified a 257-bp amplicon within the *Olfr16* mouse gene and a 187-bp amplicon spanning the edited *Hhex* mouse region from 100 ng gDNA. These amplifications were made using 32-bp barcoded forward and reverse primers (20-bp homology + 12-bp unique nucleotide sequence; Table S1). PCR was performed using high fidelity AccuStart II Taq DNA Polymerase (#95141/250, Quantabio, Beverly, MA) with 0.24 μmol/l of primer in a 100^TM^ Thermal Cycler (Bio-Rad). The PCR reaction was carried out with an initial denaturation step of 95 °C for 2 min followed by 35 cycles of denaturation at 95 °C for 20 s, annealing at 60 °C (*Olfr16*) or 63 °C (*Hhex*) for 20 s, and elongation at 68 °C for 20 s, with a final elongation step at 68 °C for 2 min. Amplicons were cleaned up using the QIAquick PCR Purification Kit (#28104, Qiagen, Inc., Germantown, MD). After elution, each sample was submitted for Sanger sequencing to verify the proper addition of the unique barcode prior to NGS (data not shown). Upon verification, the amplicons were pooled to a volume of 35 μl at a concentration of 5 ng/μl. Illumina adapters were added prior to sequencing at the University of Minnesota Genomics Center. The percent of *A* at the first SNP location and *T* at the second heterozygous SNP region in the *Olfr16* were determined from the sequencing results. After averaging those two percentages, we doubled the resulting value to obtain the final complementation value, as the 129Sv1-derived nucleotide represents only half of the donor cells present.

Additional protocols are described in Supplemental Material.

## Results

### Generation of anhepatic *Hhex* KO mice

**Fig. 1:**
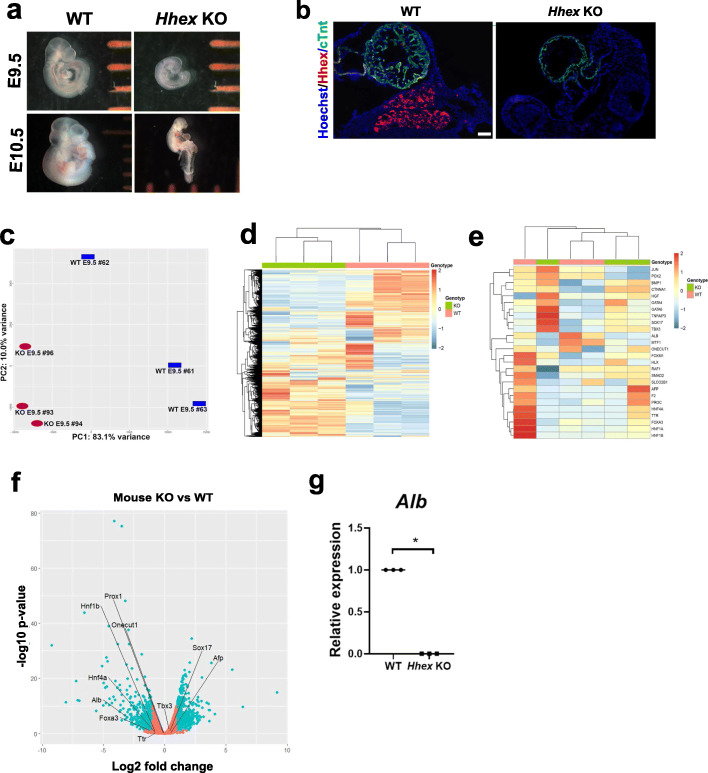
Characterization of the *HHEX* KO mouse embryos compared to WT. **a** Phenotype of the *Hhex* KO E9.5 and E10.5 mice. Separation between orange bars is 1 mm. **b** IHC images of E9.5 *Hhex* KO mouse with Hoechst (blue), anti-HHEX (red), and anti-cTnT (green) antibodies. Bars, 100 μm. **c**–**g** Gene expression analyses (RNA-seq, **c**–**f**; qRT-PC, **g**) in the *Hhex* KO E9.5 mice vs WT (*N* = 3 each group). **c** Principal component analysis. **d** Heatmap showing all the significantly up- and downregulated genes. **e** Heatmap showing liver-related genes whose expressions are significantly up- or downregulated. **f** Volcano plot showing significantly up- and downregulated genes (both in blue) and unaffected genes (orange), highlighting some liver-related genes. **g** Relative expression of transcripts. *p < 0.05

Mouse zygotes extracted from the oviduct of superovulated time-mated mice were electroporated with Cas9 and one gRNA duplexed with tracrRNA targeting exon 2 of the *Hhex* gene. E3.5 embryos were then transferred to an E2.5 pseudo-pregnant surrogate dam and extracted at 7 and 8 days later (equivalent to E9.5 and E10.5, respectively). Tail-tip genotyping of the embryos by PCR and BslI digestion identified individuals with bi-allelic knockout of *Hhex* (Supplemental Figure S2). Whole embryo images demonstrated a dramatic KO of phenotype, relative to WT at E9.5 (Fig. 1a), with retarded growth and different abnormalities as previously reported for the *Hhex* KO mouse [27]. Importantly, no viable embryos were extracted past E10.5. Immunohistochemistry of fixed-sectioned embryos at E9.5 indicated absence of liver tissue in KO embryos confirmed by loss of HHEX protein expression, while the non-liver-specific cTnT protein (used as reference) remained expressed (Fig. 1b).

Gene expression analysis of dissociated whole embryos was performed through RNA sequencing and RT-qPCR. RNA-seq data containing the expression of transcripts from three E9.5 *Hhex* KO and three WT samples (Sequence Read Archive, SRA, accession number PRJNA719797) produced a principal component analysis (PCA) graph with separation of the *Hhex* KO from WT along the first principal component (PC1) (Fig. 1c). Hierarchical clustering generated from RNA-seq reads in E9.5 embryos suggested a global dysregulation of gene expression in KO mice, relative to WT mice (Fig. 1d). With a certain heterogeneity, dysregulation of liver-specific genes was also observed in *Hhex* KO embryos, relative to WT (Fig. 1e), although when some of these genes (*Prox1, Hnf1b, Onecut1, Hnf4a, Foxa3, Ttr, Tbx3, Sox17, Afp,* and *Alb*) were annotated in a volcano plot (Fig. 1f), only *Alb* was observed as significantly downregulated in the KOs. We confirmed this result using qRT-PCR to measure *Alb* expression (*p*-value = 0.046) (Fig. 1g). Gene ontology analysis of biological processes altered in *Hhex* KO embryos identified significant differences in pathways related to organ induction, specification, and regionalization (Additional file 1). Interestingly, the greatest fold changes observed between WT and *Hhex* KO embryos were biological processes related to neural development.

### Generation of anhepatic pigs

**Fig. 2:**
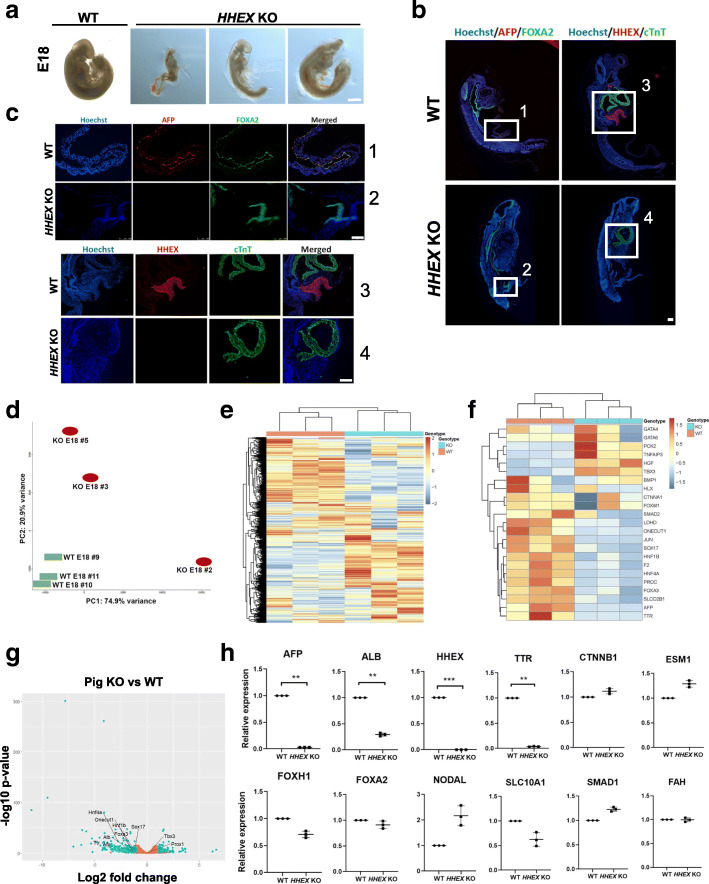
Characterization of the *HHEX* KO pig embryos compared to WT. **a** Phenotype of the *HHEX* KO E18 pigs. **b**, **c** IHC images of E18 *HHEX* KO pig embryos with Hoechst (blue), anti-AFP/HHEX (red), and anti-FOXA2/cTnT (green) antibodies. White insets in **b** correspond to numbered enlarged images in **c**. Bars: all are 100 μm, except for the bar in **a**, which is 500 μm. **d**–**h** Gene expression analyses (RNA-seq, **d**–**g**; qRT-PC, **h**) in the *HHEX* KO E18 pigs vs WT (*N* = 3 each group). **d** Principal component analysis. **e** Heatmap showing all the significantly up- and downregulated genes. **f** Heatmap showing liver-related genes whose expressions are significantly up- or downregulated. **g** Volcano plot showing significantly up- and downregulated genes (both in blue) and unaffected genes (orange), highlighting some liver-related genes. **h** Relative expression of transcripts. **p < 0.01; **p < 0.001

Microinjection of Cas9 and two gRNAs targeting exon 2 of the *HHEX* gene in single-cell activated parthenogenetic pig embryos resulted in 196 viable morulas that were transferred into two surrogate gilts (100 and 96 embryos, respectively). At E18, two and five pig embryos were collected from the uteri of those two gilts, respectively. Genotyping of the embryos by PCR and Sanger sequencing showed that they were all bi-allelic *HHEX* KO, indicated by presence of the large deletion on the *HHEX* gene, which created the expected premature stop codon in exon 3 (data not shown). These embryos showed growth retardation compared to WT E18 embryos obtained by natural mating, in addition to various developmental abnormalities, which led to lethality (Fig. 2a). Immunocytochemistry analyses on four of the *HHEX* KO embryos confirmed the absence of the liver-specific proteins HHEX and AFP, while FOXA2, an endodermal marker for epithelium of the liver bud, or heart markers such as MYL7 (not shown) and cTnT were expressed (Fig. 2b, c). RNA-seq data containing the expression of transcripts from three *HHEX* KO E18 and three WT E18 whole embryos (SRA accession number PRJNA719858) produced a PCA graph and a heatmap that clustered the *HHEX* KO samples together and separated those samples from WT, displaying the two different genotypes (Fig. 2d, e). The heatmap containing liver-specific genes demonstrated again the difference between the transcripts from the *HHEX* KO and the WT embryos, showing an opposite expression pattern between both genotypes (Fig. 2f). A volcano plot containing all the genes and highlighting some liver-related genes showed that *HNF1B, ONECUT1, HNF4A, FOXA3, TTR, SOX17, AFP,* and *ALB* were significantly downregulated in the KOs while *TBX3* and *PROX1* were not (Fig. 2g). Liver and non-liver-specific gene expression quantification by qRT-PCR in three of the *HHEX* KO embryos showed that liver-specific gene expression such as *ALB* (*p*-value = 0.003), *TTR* (*p*-value = 0.0015), *AFP* (*p*-value = 0.0028)*,* and *HHEX* (*p*-value = 0.0009) were significantly downregulated compared to the WT E18 embryos (in the case of *HHEX*, no transcripts were detected in *HHEX* KO embryos) while expressions of other genes (*FOXA2, FOXH1, NODAL, SMAD1, SLC10A1, FAH, ESM1, β-CATENIN*) were not affected (Fig. 2h). Gene Ontology (GO) pathway analysis showed that two main liver development biological processes were markedly downregulated in *HHEX* KO embryos (Additional file 1). While *S. scrofa* genome expresses 30,592 genes, our analysis showed that 308 genes were differentially regulated in the *HHEX* KO E18 embryo. Genes active during the early stages of liver organogenesis and in the liver bud were differentially expressed, while liver-specific genes such as *ALB*, *AFP*, *FOXA3*, *HNF1α,* and *SOX17* were downregulated.

### Transcriptional similarity in HHEX KO mice and pigs

**Fig. 3:**
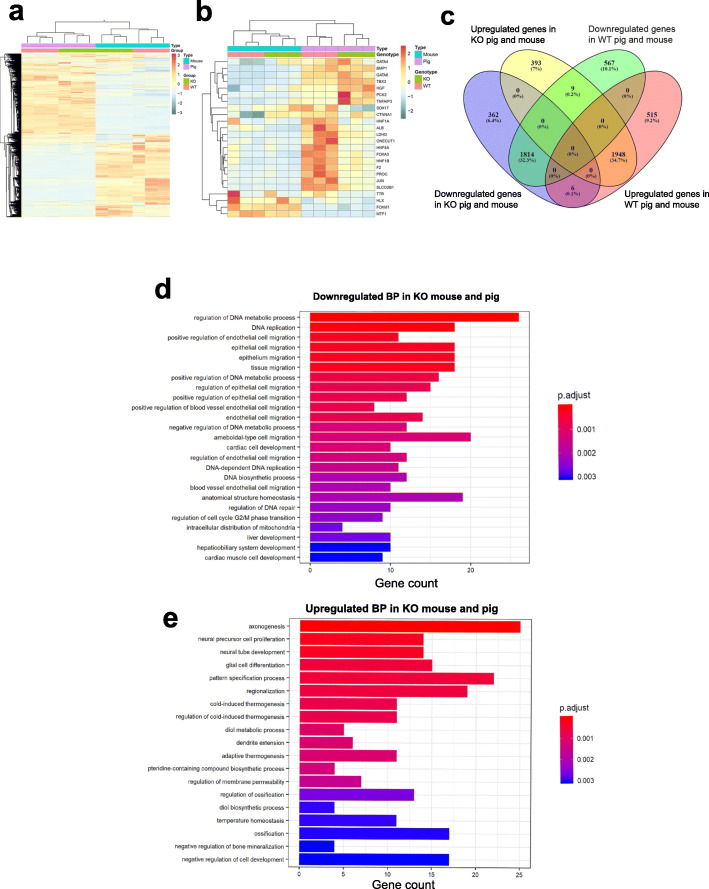
Mouse and pig RNA-seq expression data comparison. **a**, **b** Heatmaps combining mouse and pig data with all the significantly affected genes (**a**) and only those related to liver (**b**). **c** Venn diagram showing significantly down- and upregulated common genes between both species. **d**, **e** Bar plots reporting the top significantly down- (**d**) and upregulated (**e**) biological processes (BPs) related to the 362 down- and 393 upregulated common genes for both *HHEX* KO mouse and pig. The color code is proportional to significance. *N* = 6 per group

RNA-seq data from *HHEX* KO and WT samples from mice and pigs were analyzed together. The resulting comparisons (all genes or only liver-related genes) separated the two species but clustered the *HHEX* KO samples from both species next to each other, highlighting similarities between the *HHEX* KO phenotype in both mouse and pig (Fig. 3a, b). Venn diagrams indicated that the *HHEX* KO samples from both species have 362 downregulated and 393 upregulated genes in common (Fig. 3c). These genes were divided into GO pathways and the ones related to Biological Processes were the most significantly affected (Supplemental Figure S3a-b). Liver development and hepatobiliary system development were two of the GOs that appeared significantly downregulated in the *HHEX* KO from both species (Fig. 3d), and they included genes such as *AURKA, GAK, IGF2R, MET, PIK3CA, PLAU, PTN, SP1, TGFBR3,* and *XBP1* (Additional file 1). Specifically, *AURKA* is involved in liver regeneration, *GAK* in the intrahepatic bile duct development, *PLAU* in determination of liver left/right asymmetry, *IGF2R* in modulation of invasiveness of liver cells, *PTN* in the positive regulation of hepatocyte proliferation, and *SP1* positively regulates the basal transcription of *FGF21* in liver. Other GOs in this category included lymphangiogenesis (including genes *CCBE1*, *CLEC14A*, *FLT4,* and *VEGFA*), and those involved in cardiac cell and cardiac muscle cell developments (*MET, MYH10, POPDC2, SIRT1, SLC25A47, SORBS2, SEPG, TGFBR3,* and *VEGFA*), i.e., phenotypes that have been previously reported in *HHEX* KO animals. We verified these results comparing the expression levels of key genes between WT and *HHEX* KO pigs and found that *FLT4* (*p*-value < 0.0001) and *VEGFC* (*p*-value = 0.0392) but not *PROX1* (genes necessary for the lymphatic system development) were significantly downregulated in the *HHEX* KO pigs, *VEGFA* (crucial for the cardiac development) was also reduced (*p*-value = 0.0449); however, *ETV2* and *TIE2* (required for endothelial development) were unaffected (Supplemental Figure S4). The group of biological processes significantly upregulated in both *HHEX* KO embryos of both species contained several neural-related GOs (Fig. 3e). This agrees with previous findings showing that HHEX inhibits axon growth in developing neurons [40].

### Intraspecies complementation of *Hhex* KO mice

In order to determine whether the anhepatic phenotype in *Hhex* KO mice could be rescued through blastocyst complementation, eGFP-labeled miPSCs were microinjected into the blastocoel cavity of embryos previously electroporated with the mmHHEX g2.1 CRISPR/Cas9 complex. A total of 32 embryos were recovered between E9.5 and E12.5 with an eGFP^+^ signal observed in 22 (Table 1). Embryos exhibited a range of developmental phenotypes from retarded to normal growth (Figure S5a) when compared to age matched WT embryos. Importantly, more than 50% of the chimeric embryos (12 out of 22) survived beyond E10.5 which is the previously reported stage of embryonic lethality in *Hhex* KO mice.

**Table 1:** Summary of complementation experiments in mice

Recovered embryo age	Blastocysts transferred	Embryos recovered, ***n*** (%)^**a**^	GFP^**+**^ embryos, ***n*** (%)^**a,b**^
E9.5	106	17 (16)	10 (9.4, 58.8)
E10.5	14	6 (42.9)	4 (28.6, 66.7)
E11.5	32	7 (21.9)	6 (18.8, 85.7)
E12.5	81	2 (2.5)	2 (2.5, 100)

^a^Percentage of the total number of blastocysts transferred

^b^Percentage of the number of embryos recovered

**Fig. 4:**
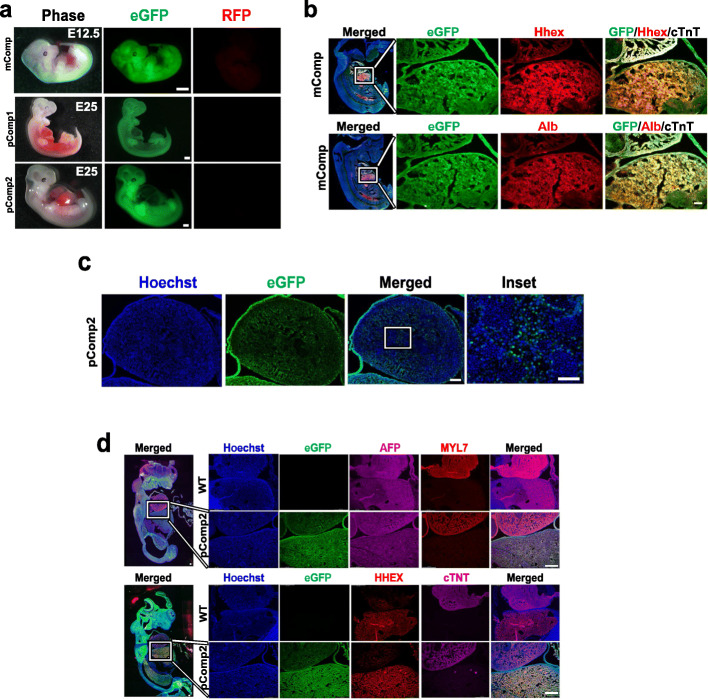
Mouse and pig complemented embryos. **a** Whole embryo images showing eGFP expression (RFP channel shown to indicate absence of signal and no autofluoresence). **b**, **d** IHC images of the mouse (**b**) and pig (**d**) complemented embryos showing the liver-heart area in the insets in the magnifications. **c** Heart area of one of the complemented pig embryos with the inset highlighting presence of both eGFP^+^ and eGFP^−^ cells within the tissue. eGFP, enhanced green fluorescent protein; RFP, red fluorescent protein; mComp, mouse complemented embryo; pComp1, pig complemented embryo 1; pComp2, pic complemented embryo 2; MYL7, myosin light chain 7; cTNT, cardiac troponin T (white signal in mouse); ALB, albumin. Bars, 1 mm in **a**, 100 μm **b** and **d** whole embryos, and in **b** magnifications; 500 μm in **d** magnifications and in **c**, except for its inset, which is 250 μm

High degrees of chimerism were present in many of the recovered complemented embryos indicated by the extensive eGFP expression that was observed, indicating widespread presence of cell progeny derived from the donor stem cells in these embryos (Fig. 4a and Supplemental Figure S5). PCR-based genotyping of tail-tip tissue was used to detect the presence of the cells carrying the KO allele but was not able to confirm whether tissue derived from the host embryos carried bi-allelic KO of *Hhex.* To answer this question, we selected 8 of the chimeric embryos (Supplemental Figure S5a-b) to amplify their *Hhex* and *Olfr16* genes with barcoded primers and performed NGS on those sequences. Because the *Olfr16* gene has SNPs that differ among the host blastocyst and the complementing donor cell line (see “Methods”), we were able to use those SNPs to quantify the percentage of sequences coming from the donor and host resulting in the degree of complementation. Sequencing of the *Hhex* gene would confirm the mutation and show which indels were produced. Complemented embryos #3, 6, and 7 were eGFP^+^ and showed both WT and KO *Hhex* bands in the gel electrophoresis following the PCR (Supplemental Figure S5b), making them the ideal target for this experiment. Our results showed that embryos #3, 6, and 7 carried 100%, 23%, and 87%, respectively, of donor cells and indicated proof of bi-allelic *Hhex* KO in #7 (Supplemental Table S4A). This embryo carried a total of 13% of *Hhex* sequences with two different mutations which accounts for the host background (Supplemental Table S4B). These results demonstrated that embryo #7 (also abbreviated “mComp” in Fig. 4a) was indeed derived from a complemented bi-allelic *Hhex* KO and comprised of 87% donor-derived cells.

Immunohistochemical analysis of *Hhex* KO embryo #7 complemented with eGFP^+^-labeled iPSCs demonstrated strong eGFP expression in the liver indicating a high proportion of donor cell derived tissue. Co-localization of eGFP signal with detection of HHEX and ALB expressions demonstrated rescue of the anhepatic phenotype and restoration of liver-specific protein expression (Fig. 4b).

### Intraspecies complementation of HHEX KO pigs

We had already demonstrated that knockout of the *HHEX* gene in pigs led to an anhepatic phenotype and we hypothesized that this liver loss might also be rescued by intraspecies complementation. To test this, we carried out SCNT to generate the KO host blastocysts and the eGFP-labeled WT donor blastomeres used for complementation. Briefly, *HHEX* KO pig zygotes were created by injecting one *HHEX* KO PEF edited in exon 2 with TALEN (Supplemental Figure S6a-b) into enucleated oocytes followed by electric fusion and activation. When these embryos reached day 4 or 5 of development, they were complemented with eGFP-labeled pig blastomeres at the same embryo stage also created by SCNT, followed by transfer to two synchronized gilts. In total, 46 and 48 complemented embryos were transferred and two E25 embryos were recovered. Whole embryo imaging showed that both embryos were highly eGFP^+^ with visible liver tissue (Fig. 4a). *HHEX* genotyping only detected *HHEX* WT sequences (data not shown), probably due to the high level of donor contribution to the chimeric embryos, although organs such as the heart were comprised of both donor-derived eGFP^+^ and host derived eGFP^−^ cells (Fig. 4c). IHC staining of the pig complemented embryos showed that HHEX expression in the liver cells was restored, in addition to the expression of other liver-related proteins, such as AFP, which co-localized with eGFP expressing cells (Fig. 4d). This confirmed that the liver tissue was derived from progeny of the WT donor cells, restoring liver organogenesis and liver associated protein expression by blastocyst complementation.

## Discussion

In the current study, we generated *HHEX* KO embryos using CRISPR/Cas9 gene editing in mouse and pig zygotes. Modifications (indels or a large deletion) in the DNA binding domain of exon 2 of *HHEX* resulted in an anhepatic phenotype that was embryonic lethal in relatively similar Carnegie stages of development in both species. Sequencing of transcripts from *HHEX* KO embryos of both species identified global dysregulation in gene expression as well as a common disruption of liver development genes. Intraspecies complementation of *HHEX* KO embryos in both the mouse and pig animal models was able to rescue the anhepatic phenotype, restoring liver organogenesis and protein expression.

Similar to previously published work, this study provides further evidence of the role of *HHEX*, particularly in liver development in the post-implantation embryo [27–29]. This study is unique, however, in that the *HHEX* KO phenotype (lack of liver and growth retardation) was shown to be similar in both mouse and pig embryos, indicating an evolutionarily conserved role of the *HHEX* gene in liver organogenesis in distinct mammalian species and an ideal target gene to produce complemented liver tissues. Analysis of transcriptome from *HHEX* KO embryos from both species identified common differentially expressed genes linked to liver and brain development. It is interesting to note that although the same region of exon 2 of *HHEX* was targeted for knockout, there were some differences between the two species, particularly in differential expression of liver-specific genes. A more robust disruption of liver development genes was observed in *HHEX* KO pig embryos, while the strongest downregulated GOs in the KO mice were associated with neuroectoderm functionalities. Even though we attempted to match developmental stages for both species based on Carnegie stages (E9.5 for mouse and E18 for pig) for transcriptomic analysis, gestation period is significantly different in both species, which results in a highly different transcription profile between the two KO species. Another explanation could be the injection of two gRNAs into pig zygotes versus one gRNA into the mouse ones, thereby increasing the frequency of loss-of-function *HHEX* alleles versus small indels generated with single guides. Intraspecies variability was also observed in the development and molecular phenotype of *HHEX* KO embryos. This could be due to the diversity of indel mutations through non-homologous end joining of CRISPR/Cas9 gene editing, in which protein structure and function could vary between individual embryos [41]. It is likely that the intraspecies variability is also due in part to the ex vivo culture of pre-implantation embryos. Whether or not this variability will alter the outcome in the blastocyst complementation setting remains to be seen, but it warrants further investigation.

A key finding of the current study was the ability to rescue liver development and liver-specific protein expression through blastocyst complementation of *HHEX* KO in both mouse and pig embryos. This observation was similar to the work of Matsunari et al. [11], in which *HHEX* KO pig embryos, generated by SCNT of TALEN edited PEFs, were complemented with huKO pig blastomeres. There are however important differences with the previous study. Here, we present a comprehensive characterization of the *HHEX* KO molecular phenotype, and the rescues described in this study were performed in two different species in which the edition of the DNA binding domain of the *HHEX* gene in the host embryo was carried out with two different technologies (CRISPR/Cas9 for mouse, TALEN for pig). Our work also differs from the Matsunari report in that the complementation in the KO mouse embryos was achieved by microinjecting eGFP^+^ miPSCs that our group had previously reprogrammed. The results reported here observed a very high contribution of donor-derived cells throughout tissues in complemented embryo, as determined by fluorescent microscopy. The cause of these observations has yet to be determined. It may be a consequence of *HHEX* function in the pre-implantation embryo or the developing organs/tissues resulting in high donor chimeric contributions from the early stage of the complemented embryogenesis, or it may be related to complementation platform methodologies, including perhaps the ability of the donor cell progeny to outcompete cells of the host.

In summary, we have developed a gene editing platform in mouse and pig embryos to efficiently knockout liver development that can then be rescued through intraspecies blastocyst complementation. This complementation strategy is the first step towards generating interspecies chimeras for the goal of producing human liver cells, tissues, or complete liver organs suitable for clinical transplantation. In this scenario, hiPSCs will be used as donor cells to complement *HHEX* KO pig embryos, and the aim is to use patient specific hiPSCs to avoid cell/tissue/organ rejection. However, the xenogeneic barrier is a major limitation that is still poorly understood and hinders progress in blastocyst complementation research. It has been suggested that both donor and host cells need to be developmentally synchronized and capable of appropriately responding to cell-to-cell signaling molecules to mitigate donor cell rejection via apoptosis after complementation [42]. Ideally, an interspecies chimeric organ will be comprised of only donor-derived cells so it might be necessary to knock out multiple genes or find one that affect other gene expressions. The fact that the lack of *HHEX* expression resulted in the downregulation of genes involved in cardiogenesis and lymphangiogenesis makes our platform a promising candidate to create not only livers derived from donor cells but also other desirable regenerated tissues. Importantly, our RNA-seq data clearly stresses the advantages of using pigs over mice as bio-incubators to generate human cells/tissues and organs for regenerative medicine, presenting a more refined liver organogenesis process enabled by longer gestation time.

## Conclusions

This work demonstrates that loss of liver development in the *HHEX* KO can be rescued via blastocyst complementation in both mice and pigs. This complementation strategy is the first step towards generating interspecies chimeras for the goal of producing human liver cells, tissues, and potentially complete organs for clinical transplantation. This technology could be the future of stem cell medicine and in this work; we have generated important proof of concepts for moving the field forward.

## Supplementary Information

**Additional file 1.** Differentially expressed genes (DEGs) and Gene Ontology (GO) pathway analysis.

**Additional file 2.** CRISPR and TALEN designs.

**Additional file 3.** KO mouse genotyping.

**Additional file 4.** RNA_seq_3.

**Additional file 5.** Endothelial.

**Additional file 6.** Mouse_mouse complementation NGS.

**Additional file 7.** TALEN edited PEFs.

**Additional file 8: Table S1.** Sequence of the barcoded primer combinations used to amplify the Olfr16 and Hhex genes of the complemented mice.

**Additional file 9: Table S2.** Sequences of the primers used in the qRT-PCR analysis.

**Additional file 10: Table S3.** List of primary and secondary antibodies used in the immunohistochemistry experiments.

**Additional file 11: Table S4.** Percentages of Orlf16 SNPs (A) and Hhex indels (B) in the mouse:mouse complemented embryos analyzed by NGS.

**Additional file 12.** Material and methods.

## Data Availability

The RNA-seq libraries are deposited in the Sequence Read Archive database of the NCBI with accession numbers PRJNA719797 (mouse data) and PRJNA719858 (pig data).

## Abbreviations

KO: Knockout
PSCs: Pluripotent stem cells
NASH: Nonalcoholic steatohepatitis
hiPSCs: Human induced pluripotent stem cells
mHTF: Modified Human Tubal Fluid media
miPSCs: Mouse induced pluripotent stem cells
IVF: In vitro fertilized
PZM-3: Porcine zygote medium-3
SCNT: Somatic cell nuclear transfer
PEF: Porcine embryonic fibroblast
BSA: Bovine serum albumin
NGS: Next Generation Sequencing
AFP: Alpha fetoprotein
SNPs: Single nucleotide polymorphisms
PCA: Principal component analysis
GO: Gene Onthology
