# Supplementary material for: Liver development is restored by blastocyst complementation of HHEX knockout in mice and pigs
Source: Stem Cell Res Ther. 2021 May 19;12:292. doi: 10.1186/s13287-021-02348-z (PMC8132445; doi:10.1186/s13287-021-02348-z)
Supplement: Supplementary file 8 — Additional file 8: Table S1. Sequence of the barcoded primer combinations used to amplify the Olfr16 and Hhex genes of the complemented mice. [file 13287_2021_2348_MOESM8_ESM.docx]

**Table S1**: Sequence of the barcoded primer combinations used to amplify the *Olfr16* and *Hhex* genes of the complemented mice.

|  | ***Olfr16*** | | | | ***Hhex*** | | | |
| --- | --- | --- | --- | --- | --- | --- | --- | --- |
| **Samples** | **Forward Primer** | **12-bp Barcode + 20-bp Homology Arm** | **Reverse Primer** | **12-bp Barcode + 20-bp Homology Arm** | **Forward Primer** | **12-bp Barcode + 20-bp Homology Arm** | **Reverse Primer** | **12-bp Barcode + 20-bp Homology Arm** |
| #1 | **mmOlfr16 NGS F1-BC1** | ATAAGGATCCGGTGGCAAGCTCAGAGACTGTG | **mmOlfr16 NGS R1-BC1** | TCGGGAAGGTCCCAAGGCACACATTCCCTTGC | **mmHhex NGS F1-BC1** | ATCTAGCCGGCCTTGCGTTCTGGATCGTGTCA | **mmHhex NGS R1-BC8** | AGCTCAGTCCGGCTCACTTGACCGCCTTTCCT |
| #2 | **mmOlfr16 NGS F1-BC2** | CTCCGATCGTCCTGGCAAGCTCAGAGACTGTG | **mmOlfr16 NGS R1-BC2** | GATTTAAGGGCCCAAGGCACACATTCCCTTGC | **mmHhex NGS F1-BC2** | TATCTCTTCCTTTTGCGTTCTGGATCGTGTCA | **mmHhex NGS R1-BC9** | ACTGCCGCGTCCCTCACTTGACCGCCTTTCCT |
| #3 | **mmOlfr16 NGS F1-BC3** | CGCCTGATCCGGTGGCAAGCTCAGAGACTGTG | **mmOlfr16 NGS R1-BC3** | AGAACTGACCTTCAAGGCACACATTCCCTTGC | **mmHhex NGS F1-BC3** | TAGATGCCGTCCTTGCGTTCTGGATCGTGTCA | **mmHhex NGS R1-BC10** | TTACTTCTCCGGCTCACTTGACCGCCTTTCCT |
| #4 | **mmOlfr16 NGS F1-BC4** | AGAATATCGTCCTGGCAAGCTCAGAGACTGTG | **mmOlfr16 NGS R1-BC4** | TGATGCAACCGGCAAGGCACACATTCCCTTGC | **mmHhex NGS F1-BC4** | CGCTCCTTCCTTTTGCGTTCTGGATCGTGTCA | **mmHhex NGS R1-BC11** | TGACGCGCGGCCCTCACTTGACCGCCTTTCCT |
| #5 | **mmOlfr16 NGS F1-BC5** | GCGGAGATCCGGTGGCAAGCTCAGAGACTGTG | **mmOlfr16 NGS R1-BC5** | GGCGGTTGGGCCCAAGGCACACATTCCCTTGC | **mmHhex NGS F1-BC5** | CGAGCGCCGTCCTTGCGTTCTGGATCGTGTCA | **mmHhex NGS R1-BC12** | AATGATCTCCGGCTCACTTGACCGCCTTTCCT |
| #6 | **mmOlfr16 NGS F1-BC6** | CATGGCCGGTCCTGGCAAGCTCAGAGACTGTG | **mmOlfr16 NGS R1-BC6** | ACTACCAACCGGCAAGGCACACATTCCCTTGC | **mmHhex NGS F1-BC6** | TCGAGAGTCCGGTTGCGTTCTGGATCGTGTCA | **mmHhex NGS R1-BC13** | AAGCGTACGTCCCTCACTTGACCGCCTTTCCT |
| #7 | **mmOlfr16 NGS F1-BC7** | GTCACGTACCTTTGGCAAGCTCAGAGACTGTG | **mmOlfr16 NGS R1-BC7** | AATAATTGGTCCCAAGGCACACATTCCCTTGC | **mmHhex NGS F1-BC7** | GTCATCGCGTCCTTGCGTTCTGGATCGTGTCA | **mmHhex NGS R1-BC14** | GTACTGATCCTTCTCACTTGACCGCCTTTCCT |
| #8 | **mmOlfr16 NGS F1-BC8** | CCTGTTGACCGGTGGCAAGCTCAGAGACTGTG | **mmOlfr16 NGS R1-BC8** | GTCGTCAACCGGCAAGGCACACATTCCCTTGC | **mmHhex NGS F1-BC8** | CCGTCTCTCCGGTTGCGTTCTGGATCGTGTCA | **mmHhex NGS R1-BC15** | GGATATACGGCCCTCACTTGACCGCCTTTCCT |
