# Supplementary material for: Liver development is restored by blastocyst complementation of HHEX knockout in mice and pigs
Source: Stem Cell Res Ther. 2021 May 19;12:292. doi: 10.1186/s13287-021-02348-z (PMC8132445; doi:10.1186/s13287-021-02348-z)
Supplement: Supplementary file 9 — Additional file 9: Table S2. Sequences of the primers used in the qRT-PCR analysis. [file 13287_2021_2348_MOESM9_ESM.docx]

**Table S2**: Sequences of the primers used in the qRT-PCR analysis.

| **Name** | **Sequence (5'-3')** | **Tm (**°C) | **Amplicon length** |
| --- | --- | --- | --- |
| mmAlb qF1 | GGCCATGTGCACCTCCTTTA | 58.5 | 194 bp |
| mmAlb qR1 | TCACACCATCAAGCTTCGGG |  |  |
| mmHprt qF1 | CAGTCCCAGCGTCGTGATTA | 58.5 | 168 bp |
| mmHprt qR1 | TGGCCTCCCATCTCCTTCAT |  |  |
| ssAFP qF1 | GCATATGGAATAGCTTCCATATTGG | 61 | 114 bp |
| ssAFP qR1 | TTCCTTGTAAGTGGCTTCTTGA |  |  |
| ssALB qF1 | CGAGATACATACAAGAGTGAAATTGC | 61 | 104 bp |
| ssALB qR1 | CATTGCTGGAGATGCTGAGA |  |  |
| ssbcatenin qF1 | AGTGGCTGATATTGATGGACAG | 61 | 94 bp |
| ssbcatenin qR1 | GATCTGCATGCCCTCATCTAA |  |  |
| ssESM1 qF1 | ATCTGCAAAGACTGTCCCTAC | 61 | 132 bp |
| ssESM1 qR1 | AGCCTTGGCTACTGAGTATTG |  |  |
| ssFAH qF1 | CTACAGGCAACTCAAGACCG | 61 | 86 bp |
| ssFAH qR1 | CCCAGTGAAGAGGTGCTTTAT |  |  |
| ssFOXA2 qF2 | AGCAGCTACTATGCCGAG | 61 | 84 bp |
| ssFOXA2 qR2 | GTAAGTGTTCATCCCGTTCATC |  |  |
| ssFOXH1 qF1 | TGGCCCAGATCATCCGT | 61 | 102 bp |
| ssFOXH1 qR1 | ATCGGTTGGAGGAGAGGTT |  |  |
| ssHHEX qF1 | GAGAGGCAGGTCAAAACCT | 61 | 123 bp |
| ssHHEX qR1 | CCTCTGGTCACAGGAATTGT |  |  |
| HPRT1 qF1 | GAAGAGCTACTGTAATGACCAGTCAACGG | 61 | 285 bp |
| HPRT1 qR1 | TCATTGTAGTCAAGGGCATAGCCTACC |  |  |
| ssNODAL qF1 | CCCAAGCAGTACAATGCCTAT | 61 | 103 bp |
| ssNODAL qR1 | GTTTCAGCAGACTCTGGATGT |  |  |
| ssSCL10A1 qF1 | ACCTCAGCATCATGATGACC | 61 | 80 bp |
| ssSCL10A1 qR1 | CCTGGAGTAAAGGTATAGGAGGA |  |  |
| ssSMAD1 qF1 | AGAGTCCCGTTCTTCCTCC | 61 | 111 bp |
| ssSMAD1 qR1 | GTGGCATGTGAGGCTCATT |  |  |
| ssTTR qF1 | TTTGCCTTGGGGAAAACCA | 61 | 117 bp |
| ssTTR qR1 | AAGTGCCTTCCAGTAGGATTTG |  |  |
| ssVEGFA qF1_qF2 | ATGCCAAGTGGTCCCAG | 61 | 107 bp |
| ssVEGFA qR1 | CTCGATTGGACGGCAGTAG |  |  |
| ssFLT4 qF1_qF2 | GTGAGAGACCCGGAGCA | 62 | 108 bp |
| ssFLT4 qR1 | GATGTTGAGGCCAGGGATG |  |  |
| ssPROX1 qF1_qF2 | CGGTCCCGGGATTCTTG | 62 | 130 bp |
| ssPROX1 qR1 | ATGTCAACTCTTCTCCTCTTGG |  |  |
| ssVEGFC qF1_qF2 | GCGAGGCCAAGGCTTAT | 62 | 141 bp |
| ssVEGFC qR1 | TATGTTGCCAGCCTCCTTTC |  |  |
| ssTIE2 qF3 | AACAAGCTTCCTTCCTACCAG | 62 | 140 bp |
| ssTIE2 qR3 | GGGCACCGAATGGATGAA |  |  |
| ssETV2 qF4 | AGTTCCAACTGTGCGACC | 60 | 82 bp |
| ssETV2 qR4 | CAGCTTCTCATAATTCATGCCG |  |  |
