# Supplementary material for: Liver development is restored by blastocyst complementation of HHEX knockout in mice and pigs
Source: Stem Cell Res Ther. 2021 May 19;12:292. doi: 10.1186/s13287-021-02348-z (PMC8132445; doi:10.1186/s13287-021-02348-z)
Supplement: Supplementary file 10 — Additional file 10: Table S3. List of primary and secondary antibodies used in the immunohistochemistry experiments. [file 13287_2021_2348_MOESM10_ESM.docx]

**Table S3**: List of primary and secondary antibodies used in the immunohistochemistry experiments.

|  | **Antibody** | **Vendor** | **Catalog #** | **Resource**  **Identifier**  **(RRID)** | **Dilution** | **Used in IHC for:** |
| --- | --- | --- | --- | --- | --- | --- |
| **Primary** | Rabbit anti-ALBUMIN | Sigma-Aldrich, St. Louis, MO | A3293 | AB_258063 | 1:500 | Mouse |
|  | Mouse anti-cTnT (13-11) | Invitrogen, Waltham, MA | MA5-12960 | AB_11000742 | 1:200 | Mouse and pig |
|  | Goat anti-FOXA2 | R&D Systems, Minneapolis, MN | AF2400 | AB_2294104 | 1:200 |  |
|  | Rabbit anti-HHEX | R&D Systems, Minneapolis, MN | MAB83771 | N/A | 1:20 |  |
|  | Chicken anti-eGFP | Abcam, Cambridge, MA | ab13970 | AB_300798 | 1:500 |  |
|  | Mouse anti-AFP (39) | Santa Cruz Biotechnology, Dallas, TX | sc130302 | AB_2223934 | 1:200 | Pig |
|  | Rabbit anti-MYL7 | Sigma-Aldrich, St. Louis, MO | HPA013331 | AB_1854245 | 1:200 |  |
| **Secondary** | Alexa Fluor 488 Donkey anti-mouse | Thermo Fisher Scientific, Waltham, MA | A32766 | AB_2762823 | 1:1000 | Mouse |
|  | Alexa Fluor 488 Donkey anti-Rabbit | Thermo Fisher Scientific, Waltham, MA | A32790 | AB_2762833 | 1:1000 |  |
|  | Alexa Fluor 555 Donkey anti-Rabbit | Thermo Fisher Scientific, Waltham, MA | A21428 | AB_141784 | 1:1000 |  |
|  | Alexa Fluor 555 Donkey anti-Goat | Thermo Fisher Scientific, Waltham, MA | A32816 | AB_2762839 | 1:1000 |  |
|  | Alexa Fluor 594 donkey anti-Goat | Jackson ImmunoResearch Laboratories, Inc., West Grove, PA | 705-585-003 | AB_2340432 | 1:200 | Pig |
|  | Alexa Fluor 488 donkey anti-Chicken | Jackson ImmunoResearch Laboratories, Inc., West Grove, PA | 703-545-155 | AB_2340375 | 1:200 |  |
|  | Alexa Fluor 594 donkey anti-Rabbit | Jackson ImmunoResearch Laboratories, Inc., West Grove, PA | 711-585-152 | AB_2340621 | 1:200 |  |
|  | Alexa Fluor 647 donkey anti-Mouse | Jackson ImmunoResearch Laboratories, Inc., West Grove, PA | 715-605-151 | AB_2340863 | 1:200 |  |
