# Supplementary material for: Liver development is restored by blastocyst complementation of HHEX knockout in mice and pigs
Source: Stem Cell Res Ther. 2021 May 19;12:292. doi: 10.1186/s13287-021-02348-z (PMC8132445; doi:10.1186/s13287-021-02348-z)
Supplement: Supplementary file 11 — Additional file 11: Table S4. Percentages of Orlf16 SNPs (A) and Hhex indels (B) in the mouse:mouse complemented embryos analyzed by NGS. [file 13287_2021_2348_MOESM11_ESM.docx]

**Table S4:** Percentages of *Orlf16* SNPs (A) and *Hhex* indels (B) in the mouse:mouse complemented embryos analyzed by NGS.

A)

| ***Orlf16*** | **#1** | **#2** | **#3** | **#4** | **#5** | **#6** | **#7** | **#8** |
| --- | --- | --- | --- | --- | --- | --- | --- | --- |
| **Position 56 (A%)** | 52 | 22 | 52 | 52 | 52 | 11 | **43** | 1 |
| **Position 134 (T%)** | 52 | 24 | 52 | 51 | 53 | 13 | **44** | 3 |
| **Average SNPs (%)** | 52 | 23 | 52 | 51 | 52 | 12 | **44** | 2 |
| **Donor contribution (%)** | 100 | 46 | 100 | 100 | 100 | 23 | **87** | 4 |

B)

| ***Hhex* in #7** | **Reference** | **Found** | **%** | **Result** |
| --- | --- | --- | --- | --- |
| **Position 115** | CCTTGCT | C | 7 | 6-bp deletion |
| **Position 118** | T | TG | 6 | 1-bp insertion |
| **Host contribution (%)** |  |  | **13** |  |
