# Supplementary material for: Liver development is restored by blastocyst complementation of HHEX knockout in mice and pigs
Source: Stem Cell Res Ther. 2021 May 19;12:292. doi: 10.1186/s13287-021-02348-z (PMC8132445; doi:10.1186/s13287-021-02348-z)
Supplement: Supplementary file 12 — Additional file 12. Material and methods. [file 13287_2021_2348_MOESM12_ESM.docx]

**Supplemental material**

**Material and methods**

All research involving mice was approved by the University of Minnesota Institutional Animal Care and Use Committee (1910-37539A). Mice had a light cycle of 12:12-h light:dark cycle, with lights on at 7:00 a.m. Surrogate female mice were fed Teklad 2919 diet and all other mice were fed Teklad 2918 diet. Mice were housed in individually ventilated micro-isolator cages on corn-cob bedding and enviro-dri enrichment.

The research involving pigs was performed in Recombinetics, Inc. facilities under its Animal Welfare Assurance #A4728-01, and all the protocols were reviewed and approved by the Recombinetics Institutional Animal Care and Use Committee (RCI-1805-07A). Pigs were housed in individual crates in a temperature-controlled room (25^o^C). Pigs were given access to water freely and were provided with commercial feed (Form-a-feed, Inc., New Richmond, WI), twice per day. The health condition of all pigs was observed daily at feeding by the animal husbandry.

***Mouse Induced Pluripotent Stem Cells (miPSCs)***

The miPSC line *UMN-3F10* used in this study was previously generated in our laboratory (1). The miPSCs were cultured at 37^o^C, 5% CO_2_ on irradiated mouse embryonic fibroblasts (#PSC001, R&D Systems, Inc., Minneapolis, MN) in miPSC medium consisting of knockout DMEM with 4.5 g/l D-glucose and sodium pyruvate (#10829018, Thermo Fisher Scientific), 10% Gibco^TM^ Knockout^TM^ serum replacement (#A3181501, Thermo Fisher Scientific, Waltham, MA), 10% fetal bovine serum (#SH30071.02HI, HyClone, Logan, UT), 1X Gibco^TM^ MEM non-essential amino acids (#11140050, Thermo Fisher Scientific), 1X Gibco^TM^ GlutaMAX (#35050061, Thermo Fisher Scientific), 0.1 mM 2-mercaptoethanol (#31350010, Thermo Fisher Scientific), 1X Corning^TM^ penicillin/streptomycin (#30002CI, Thermo Fisher Scientific), and 1,000 U/ml ESGRO-LIF (#ESG1107, Millipore Sigma, Burlington, MA). *UMN-3F10* cells constitutively express transmembrane bound eGFP.

***Preparation of miPSCs for blastocyst injection***

miPSC cultures were washed in 1X PBS without calcium or magnesium (#14190144, Thermo Fisher Scientific), then exposed to 0.1 ml/cm^2^ StemPro Accutase^TM^ cell dissociation Reagent (#A1110501, Thermo Fisher Scientific) for 5 min at 37^o^C. The cell suspension was then removed from the plate and diluted with 2 volumes of miPSC media and centrifuged for 5 min at 328 x g. The cells were re-suspended in 5 ml of PBS and centrifuged again for 3 min at 328 x g and re-suspended in miPSC media. Feeder cells were depleted from the cell suspension by adherent culture in a T75 flask for 1.5 h at 37^o^C, 5% CO_2_. After fibroblast depletion, the remaining cell suspension was collected and centrifuged for 5 min at 328 x g. The miPSCs were re-suspended in mouse iPSC media, transferred to a 1.7 ml micro-centrifuge tube, placed on ice for transport to the injection suite, and injected within the next 2 h. Cells were used between passage 19 and 25.

***Mouse embryo extraction***

To isolate post-implantation embryos, pregnant mice were euthanized via carbon dioxide asphyxiation followed by cervical dislocation around 10 a.m. at either E9.5, E10.5, E11.5, or E12.5. Uteri were quickly dissected out and placed in ice-cold Hanks Balanced Salt Solution (HBSS; #14175095, Thermo Fisher Scientific) then further dissected in HBSS filled petri dish on ice to isolate conceptuses. They were transferred to a fresh petri dish on ice with HBSS to extract embryos under a stereomicroscope. After, whole embryo images were taken, and the tip of the tail was separated for genotyping.

***Mouse immunohistochemistry***

Mouse embryos (either WT, *Hhex* Knockout (KO), or complemented) were harvested at E9.5, E10.5, E11.5 and E12.5 and immersion-fixed with fresh 4% paraformaldehyde (PFA; #J19943K2, Thermo Fisher Scientific) in PBS at room temperature (RT) for 1.5 or 2.5 h for younger and older embryos, respectively. Embryos were rinsed overnight in PBS and then cryoprotected by immersion in increasing concentrations of sucrose in PBS up to 30%. After cryoprotection (a maximum of 2 days after harvest), sucrose was removed from the embryos by dabbing with a paper towel, and samples were embedded in Tissue Freezing Medium (#TFM-C, General Data Co., Inc., Cincinnati, OH) and snap-frozen on dry ice. Cryosections of 10 μm thickness were cut on a Leica cryostat (Wetzlar, Germany). Slides were stored at -20°C until the day of staining. Frozen sections were placed on a heating block for ½ hour before antibody staining. Prior to immunohistochemical staining, antigen retrieval was performed using boiling sodium citrate buffer (10 mM sodium citrate acid, 0.05% Tween 20, pH 6). Sections were rinsed with PBS, followed by PBS with 0.025% Tween 20 (PBST). Tissue was permeabilized with PBST containing 0.1% Triton X-100 for 10 min. Nonspecific binding was blocked with 10% normal horse serum (#H0146-5ML, Sigma-Aldrich, St. Louis, MO) in PBS. Sections were incubated with primary antibodies overnight at 4°C, then incubated with secondary antibodies for 2 h at RT (see antibody information in Table S3), and counterstained with 10 μg/ml Hoechst 33342 (#H3570, Invitrogen^TM^, Waltham, MA) for 10 min at RT.

***Pig embryo collection, processing, and immunohistochemistry analysis***

The day of the collection, E18 or E25 embryos were flushed from the uteri using cold 1X PBS. After taking whole embryo images and separating the tip of the tail for genotyping, they were fixed in 4% PFA for 1 h at 4°C. Then, embryos were washed three times with 1X PBS and cryopreserved with a sucrose-in-PBS gradient (5%, 15%, 30%) for 2 h each, at 4°C. The embryos were embedded in Tissue Tek**^®^** Optimum Cutting Temperature (OCT) solution (#4583, Sakura Finetek USA, Inc., Torrance, CA), snap- frozen on dry ice, and stored at -80°C until the day of sectioning. Each embryo was cryo-sectioned sagitally in 10 µm sections in a Leica cryostat for immunocytochemistry staining. This protocol was performed as follows: slides containing embryo sections were washed with 1X PBS three times for 10 min at RT, permeabilized with 0.1% Triton X-100 in PBST for 10 min at RT, blocked with 5% Normal Donkey Serum (#S30-100ML, Millipore Sigma) in PBST for 1 h at RT, incubated with primary antibody in blocking solution at 4°C overnight (see antibody information in Table S1). The following day they were washed with PBST three times for 10 min at RT, incubated with secondary antibody in blocking solution at RT for 1 h, counterstained with 10 μg/ml Hoechst 33342 (#H3570, Invitrogen^TM^) in 1X PBS for 10 min at RT, and washed with PBST three times for 10 min at RT and mounted with Immu-Mount Shandon (#9990402, Thermo Fisher Scientific). The staining was visualized with a Leica DM6000B microscope and pictures were taken with an attached Leica DFC7000T camera.

***HHEX PCR genotyping***

On the day of embryo collection, a small piece of embryo tail was removed using clean scissors and forceps and placed into a PCR strip tube labeled with animal and embryo number. To extract the genomic DNA (gDNA), 200 μl of lysis buffer (10 mM Tris-Cl pH 8.0, 2 mM EDTA, 2.5% Tween 20, 2.5% Triton-X 100, 100 mg/ml Proteinase K) was added to each tail piece and digested at 50°C for 60 min followed by 95°C for 15 min. gDNA concentration and quality were measured with the Nanodrop 2000c Spectrophotometer (Thermo Fisher Scientific). *HHEX* PCR was performed with mouse (Forward 5’-GCTGGTCTGGGTGGTAGAAC-3’; Reverse: 5’-AGAAAGTCCCAAATCCCGGG-3’) and pig (Forward 5’-GCGTGTGACTTTGGGTGTTC-3’; Reverse: 5’-CTCCAAATCTCACTCCGGCC-3’) *HHEX* primers and a AccuStart II GelTrack PCR SuperMix (#95136-100, Quantabio, Beverly, MA) in a 100^TM^ Thermal Cycler (Bio-Rad, Hercules, CA). PCR conditions were as follows: an initial denaturation at 95°C for 3 min, 35 cycles of 95°C for 20 s, 62°C for 45 s, and 72°C for 30 s, and a final step of 72°C for 5 min. Because only one guide RNA was used in the mouse *Hhex* edition, the mouse PCR product (548 bp) required digestion with BslI (#R0555, New England BioLabs, Inc., Ipswich, MA) for 2 h at 55°C to confirm the edition. Digested WT products resulted in four bands (208 + 286 + 48 + 15 bp) while digested *Hhex* KO products yielded three bands (494 + 48 + 15 bp). The diagnosis of the pig embryos edited with TALEN also required digestion of the PCR product with HindIII (#R0104, New England BioLabs, Inc.) for 2 h at 37°C. Digestion of the *HHEX* KO product resulted in 174 bp and 234 bp bands whereas the 408-bp *HHEX* WT amplicon did not digest).

***Total RNA extraction, complementary DNA (cDNA) synthesis and qRT-PCR***

Whole embryos were homogenized using 1 ml Trizol (#15596026, Thermo Fisher Scientific) and incubated in it for 5 min at RT. Then, 200 µl of chloroform was added and mixed thoroughly by shaking the tubes 15 times. After a 3 min incubation at RT, the samples were centrifuged at 1200 x g for 20 min at 4°C. The aqueous phase was transferred to a new tube and one volume of 70% ethanol was added and mixed thoroughly. The sample was loaded into a spin column (PureLink^TM^ RNA Mini Kit, #12183018A, Thermo Fisher Scientific) and centrifuged at 1200 x g for 15 sec at RT. From here, the protocol from that kit was followed, and the total RNA was stored at -80°C. cDNA was synthesized from 500 ng of total RNA using the qScript cDNA Synthesis Kit (#95047, Quantabio) and following the manufacturer’s instructions. Gene expression analyses were performed amplifying liver (*HHEX*, *AFP*, *ALBUMIN-ALB*, *TRANSTHYRETIN-TTR*) and non-liver-specific (*BETA-CATENIN*, *ESM1*, *FAH*, *FOXH1*, *FOXA2*, *NODAL*, *SLC10A1*, *SMAD1*) transcripts using 1.5 ng of cDNA, 2X Precision Melt Supermix (#172-5112, Bio-Rad) and 2.5 µM of each forward and reverse primer (Table S2) in a final volume of 20 µl. The *HPRT1* gene was used as a housekeeping marker. qRT-PCR assays were run in a CFX Connect Real-Time PCR Detection System (Bio-Rad) with the following amplification conditions: an initial denaturation at 95°C for 3 min; 40 cycles of 95°C for 10 sec, 60°C for 30 sec, 72°C for 30 sec; and a final Melting Curve ramp. The results were analyzed by the ΔΔC_t_ method, and the statistical analyses (Shapiro-Wilk and Unpaired t-tests) and graphs performed with GraphPad Prism (version 8.2.0 for Windows, GraphPad Software, San Diego, CA, [www.graphpad.com](http://www.graphpad.com)”).

***Bioinformatic analyses***

**Mouse-mouse complementation:**

Demultiplexing was performed using MIGEC pipeline (v1.2.9) (2). During this step, fastq files were separated for each barcoded sample and sample barcodes were removed for further analysis. The number of nucleotides in certain positions was counted using Biopython (v1.77, Biopython.org). Bowtie2 (v.2.4.2) (3) was used to align the sequencing reads to the reference sequence. For each sample, aligned sequences in BAM format produced by bowtie2 (v.2.4.2) were used to call INDELs using SAMtools (v.1.10) mpileup (4), and the BCFtools (v.1.10.2) (4) were then used to filter the SNPs and generated a multi-sample variant call format (VCF) file.

**RNA-seq data:**

RNA-seq was performed on the RNA extracted from E9.5 WT mouse-, E18 WT pig-, E9.5 *Hhex* KO mouse- and E18 *HHEX* KO pig-whole embryos (n=3 each group). Sequencing was performed on the NextSeq Mid platform using pair-end reads at a read length of 76 nucleotides. An average of 29.21 million reads (24.9 ~ 31.9 million) were generated per library, with an average quality score passing quality filter above Q30. The demultiplexed FASTQ files were analyzed using a customized pipeline (CHURP; <https://github.com/msi-ris/CHURP>) developed and maintained by the University of Minnesota Supercomputing Institute (MSI). Briefly, FastQC v0.11.7 (http://www.bioinformatics.babraham.ac.uk/projects/fastqc/) was used to confirm the sequencing quality of the FASTQ files. Then adapters and low-quality reads were trimmed using Trimmomatic v0.33 (<http://www.usadellab.org/cms/index.php?page=trimmomatic>) (5). An additional quality check with FastQC was performed on the post-trimming sequences to ensure successful adaptor and quality trimming. The remaining sequences were then aligned to the reference genome (GRCm38/mm10 and Sscrofa10.2/susScr3) using HISAT2 v2.1.0 (https://ccb.jhu.edu/software/hisat2/index.shtml) and transcript abundance was counted using subread v1.6.2 (<http://subread.sourceforge.net/>) (6, 7). Differential gene expression analyses between KOs and WTs of each species were performed in R v3.6.2 using *edgeR* package (<https://bioconductor.org/packages/release/bioc/html/edgeR.html>) (8), with cutoffs of p-value <0.05 and fold change >2, and the results were visualized with a volcano plot using ggplot2 (version 3.3.3; https://cran.r-project.org/web/packages/ggplot2/index.html/) in R 3.6.2. In the volcano plot, the X axis represents a log2 (fold change) and the Y axis represents -log10 (p-values). The blue indicates genes with a threshold p-value < 0.05 and an absolute value of fold change > 2, and the red indicates genes with a threshold either p-value > 0.05 or an absolute value of fold change < 2. The key genes in liver development were labeled using the R package ‘ggrepel’ (version 0.9.1; https://cran.r-project.org/web/packages/ggrepel/index.html).

Gene ontology analysis of differentially expressed genes was performed by functionally annotating the genes and performing overrepresentation enrichment testing using PANTHER (<http://pantherdb.org/>) (9). Heatmaps were generated using the log transformed counts with *pheatmap* v1.0.12 (https://cran.r-project.org/web/packages/pheatmap/index.html) packages. Hierarchical clustering was performed using average linkage clustering method with correlation coefficient as similarity metric.

**List of abbreviations**

miPSCs, mouse induced pluripotent stem cells; KO, knockout; PFA, paraformaldehyde; RT, room temperature; PBST, PBS with 0.025% Tween 20; OCT, Optimum Cutting Temperature; gDNA, genomic DNA; cDNA, complementary DNA.

**References**

1. Greder L V., Gupta S, Li S, Abedin MJ, Sajini A, Segal Y, et al. Analysis of endogenous Oct4 activation during induced pluripotent stem cell reprogramming using an inducible Oct4 lineage label. Stem Cells. 2012;30(11):2596-2601.

2. Shugay M, Britanova O V, Merzlyak EM, Turchaninova MA, Mamedov IZ, Tuganbaev TR, et al. Towards error-free profiling of immune repertoires. Nat Methods. 2014;11(6):653-655.

3. Langmead B, Salzberg SL. Fast gapped-read alignment with Bowtie 2. Nat Methods. 2012;9(4):357-359.

4. Li H, Handsaker B, Wysoker A, Fennell T, Ruan J, Homer N, et al. The Sequence Alignment/Map format and SAMtools. Bioinforma Appl NOTE. 2009;25(16):2078-2079.

5. NBolger AM, Lohse M, Usadel B. Trimmomatic: A flexible trimmer for Illumina sequence data. Bioinformatics. 2014;30(15):2114-2120.

6. Kim D, Langmead B, Salzberg SL. HISAT: A fast spliced aligner with low memory requirements. Nat Methods. 2015;12(4):357-360.

7. Liao Y, Smyth GK, Shi W. featureCounts: an efficient general purpose program for assigning sequence reads to genomic features. Bioinformatics. 2014;30(7):923-930.

8. Robinson MD, McCarthy DJ, Smyth GK. edgeR: A Bioconductor package for differential expression analysis of digital gene expression data. Bioinformatics. 2009;26(1):139-140.

9. Mi H, Muruganujan A, Thomas PD. PANTHER in 2013: Modeling the evolution of gene function, and other gene attributes, in the context of phylogenetic trees. Nucleic Acids Res. 2013;41(D1).

**Legends for supplemental figures**

**Supplemental Figure S1**. CRISPR and TALEN designs used to create the KO *HHEX* mouse (**a:** CRISPR) and pig embryos (**b:** CRISPR; **c:** TALEN).

**Supplemental Figure S2.** PCR *Hhex* genotyping results of 10 mouse embryos electroporated with mmHhex g2.1, tracrRNA and Cas9. CRISPR edition in mouse #10 resulted in an indel in one of the alleles that produced a different digested band from the expected one. Digested WT PCR product: 208 + 286 + 48 + 15 bp. Digested *Hhex* mutated PCR product: 494 + 48 + 15 bp. U= undigested product; C= digested product; L: 100 bp ladder; WT: Wild Type; NTC: No template control.

**Supplemental Figure S3.** Gene expression analyses in *HHEX* KO E9.5 mouse and E18 pig compared to WT. **(a, b)** Bar plots reporting the top significantly enriched biological processes (BP), cellular components (CC) and molecular functions (MF) related to the 362 down- and 393 upregulated common genes in both KO species. The color code is proportional to significance. N= 6 per group.

**Supplemental Figure S4.** Vasculogenesis, angiogenesis and cardiogenesis in E18 WT and *HHEX* KO pig embryos. **(a)** Relative expression of *PROX1*, *FLT4* and *VEGFC* (vasculogenesis), *VEGFA* (cardiogenesis), and *TIE2* and *ETV2* (angiogenesis) transcripts in the *HHEX* KO embryos compared to the WT (WT=1). **(b)** IHC staining images with anti-FLT4 antibody. *: p < 0.05; ****: p < 0.0001. N= 3 per group. Bars in (b): 250 µm.

**Supplemental Figure S5.** Eight chimeric mouse embryos selected for NGS analysis. **(a)** Whole embryo images showing eGFP expression (RFP channel shown to indicate absence of signal and no autofluoresence). Embryos #1-6 were E11.5 and #7-8 were E12.5. **(b)** Gel electrophoresis showing *Hhex* genotyping results. Digested WT PCR product: 208 +286 + 48 + 15 bp. Digested Hhex mutated PCR product: 494 + 48 + 15 bp. Bar in (a): 1 mm. eGFP: enhanced green fluorescence protein; RFP: red fluorescent protein; U= undigested product; C= digested product; L: 100 bp ladder; WT: Wild Type; NTC: No template control.

**Supplemental Figure S6.** Genotyping of the TALEN-edited PEF cell line (*HHEX* KO) compared to WT. **(a)** Gel electrophoresis showing results of the PCR amplification of part of the *HHEX* gene followed by HindIII restriction. **(b)** Alignment of the Sanger sequencing results showing that the *HHEX* KO PEFs contain the 7-bp insertion. WT: Wild Type; NTC: no template control; PEFs: pig embryonic fibroblasts; WT *HHEX* amplicon digestion: 408 bp; *HHEX* KO amplicon digestion: 174 + 234 bp.
